# Brief Report: Single and Repeat Screening with the Modified Checklist for Autism in Toddlers-Revised in Young Children at Higher Likelihood for Autism Spectrum Disorder

**DOI:** 10.1007/s10803-023-06138-9

**Published:** 2023-10-31

**Authors:** Chandni Parikh, Sally Ozonoff

**Affiliations:** Department of Psychiatry & Behavioral Sciences, MIND Institute, University of California-Davis, Davis, CA USA

**Keywords:** Autism screening, Psychometrics, Infant siblings

## Abstract

Purpose: To compare the utility of single versus repeated autism screening in a sample at higher likelihood (HL) for ASD, following both screen positives and all screen negatives to diagnostic outcome. Methods: Using a prospective infant sibling design, the current study followed 135 toddlers at HL for ASD and conducted diagnostic evaluations on the full sample at 18, 24, and 36 months. The psychometric properties of the M-CHAT-R using both concurrent and predictive diagnostic evaluations were compared in a group screened once (at 18 months only, *n* = 60) or twice (at both 18 and 24 months, *n* = 75). The study also examined consistency in reporting of ASD symptoms across the M-CHAT-R and a developmental concerns interview, comparing the HL group to a group with lower likelihood (LL) for ASD (*n* = 88). Results: Sensitivity and specificity of the M-CHAT-R were high (75 − 95%), consistent with previous research. Positive predictive value (43 − 76%) was higher in this HL group than in previous community samples. Repeat screening improved sensitivity with little cost to specificity. At both 18 and 24 months, HL parents were more consistent in their reporting on the M-CHAT-R and a concerns interview than LL parents. Conclusion: The M-CHAT-R has strong psychometric properties when used with groups at HL for ASD, suggesting that scores over the screening cutoff of 3 should lead to prompt diagnostic evaluation referrals in children with older siblings on the spectrum.

Early identification of autism spectrum disorder (ASD) is crucial for accessing interventions that promote optimal behavioral, social, and functional outcomes. Signs of ASD manifest within the first years of life (Ozonoff et al., 2010; Zwaigenbaum et al., 2013) and a diagnosis can be reliably made by 18–24 months of age (Guthrie et al., 2013; Ozonoff et al., 2015; Pierce et al., 2019), yet the average age of ASD diagnosis in the United States remains around four years old (Maenner et al., 2023).

For over a decade, the American Academy of Pediatrics (AAP) has recommended routine developmental surveillance and ASD-specific screening for all children at 18- and 24-month well-child exams as a means to lower the diagnostic age (Hyman et al., 2020). However, the heterogeneous timing of ASD symptom emergence makes early identification a challenge (Ozonoff et al., 2018; Zwaigenbaum & Maguire, 2019), as does variable performance of screening tools, potential harm of classification errors, and unknown cost-benefit ratio of universal screening (McCarty & Frye, 2020; Yuen et al., 2018).

One of the most extensively used screening tools is the Modified Checklist for Autism in Toddlers (M-CHAT; Robins et al., 2001) and its latest revision, the M-CHAT-Revised with Follow-Up (M-CHAT-R/F; Robins et al., 2014); in this paper, we collectively refer to both versions of the instrument as the M-CHAT-R. A recent systematic review and meta-analysis reported pooled sensitivity of 83% (95% CI: 77–88%) and pooled specificity of 94% (95% CI: 89–97%) across 51 studies (Wieckowski et al., 2023).

Several factors influence the psychometric performance of the M-CHAT-R, including the type of sample (elevated likelihood versus unselected community), reporter familiarity with ASD, age at screening, number of screenings, use of follow-up interviews, time between screening and diagnosis (e.g., concurrent versus prospective identification), and ongoing surveillance of children who screen negative. Psychometric properties of sensitivity (SE), specificity (SP), and positive and negative predictive value (PPV/NPV) tend to be lower in community than clinical samples (Carbone et al., 2020; Guthrie et al., 2019; Toh et al., 2018) and in younger than older toddlers (Øien et al., 2018; Sturner et al., 2017a; Sturner, Howard, Bergmann, Stewart et al., 2017). In the few studies that have conducted repeated screenings to identify cases missed by initial screening, as recommended by the AAP, there is a large increase in SE without a compromise in SP (Wieckowski et al., 2023).

Accurate calculations of SE and SP require systematic procedures for identifying missed cases within the screen-negative sample, but in large community studies it is not practical or feasible to evaluate all screen-negative cases to confirm diagnostic status (Levy et al., 2020; McPheeters et al., 2016; Sheldrick et al., 2023). Samples that are at higher likelihood of an ASD outcome, such as younger siblings of children with ASD, can be helpful in this regard. The design of most infant sibling studies includes diagnostic evaluation of the entire sample, regardless of screening status, permitting calculation of SE and SP, in addition to PPV. Familial samples are also of interest because the psychometric properties of screeners may be different given enhanced knowledge of ASD symptoms in parents who already have an affected child. Only two studies have used the M-CHAT-R in toddlers at elevated likelihood for ASD due to family history. Bradbury et al. (2020) compared M-CHAT-R screening results of toddlers at higher likelihood for ASD (*n* = 187) to lower likelihood toddlers from the M-CHAT-R validation study (*n* = 15,848; Robins et al., 2014). The M-CHAT-R’s PPV in the elevated likelihood group (53%) was much higher than the PPV in the M-CHAT-R validation sample (14%). SE and SP could not be calculated since the lower likelihood sample was not systematically followed if they screened negative. Weitlauf et al. (2015) also used the M-CHAT-R in a sample at higher likelihood for ASD (*n* = 74) but did not provide lower likelihood comparison data. Most of the sample was followed, regardless of screening status, to later evaluation. The study reported that 18-month M-CHAT-R scores had high SE, SP, PPV, and NPV (generally above 70%) for concurrent 18-month diagnosis as well as predictive identification of ASD at 24- or 36-months. Neither study of higher likelihood infant siblings (Bradbury et al., 2020; Weitlauf et al., 2015) screened participants more than once and compared single to repeat screenings.

The goal of the current study is threefold: (1) compare psychometric properties of the M-CHAT-R in two non-overlapping samples of toddlers at higher likelihood for ASD, a single screened group (at 18 months) and a repeat screened group (at both 18 and 24 months); (2) prospectively follow *all* children, regardless of screening status, to outcome age at 36 months, to allow for calculation of SE, SP, PPV, and NPV; and (3) examine consistency in reporting of ASD symptoms across the M-CHAT-R and a developmental concerns interview in parents with higher versus lower familiarity with ASD based on family history.

## Methods

### Participants

The M-CHAT-R was completed by parents of 135 toddlers with an older sibling with ASD (Higher Likelihood or HL group) at 18 months. A subset (*n* = 75) was screened a second time at 24 months (see Table 1); this subsample was not selected based on clinical concerns or screening history. The smaller size of the repeat-screened group was due to later addition of the second screening to the study protocol, after some participants had already aged out of the 24-month screening window. HL infants had at least one older sibling with ASD (proband), whose diagnosis was confirmed using the Autism Diagnostic Observation Schedule, Second Edition (ADOS-2; Lord et al., 2012) and the Social Communication Questionnaire (SCQ; Rutter et al., 2003).

**Table 1:** HL Sample (n = 135) Demographic Characteristics

	Single Screening (18-months) *n* = 60	Repeat Screenings (18- & 24-months) *n* = 75	Statistic
**Age at screening in months, Mean (SD)**	18.23 (0.52)	18m: 18.02 (0.32) 24m: 24.08 (0.43)	*t* (133) = 2.87, *p* < .01
**Age at diagnostic evaluation in months, Mean (SD)**	36.37 (0.88)	36.36 (0.85)	*t* (133) = 0.11, *p* = .46
**Sex, n (%)**			χ^2^ = (1, *n =* 135) = 0.30, *p* = .60
Male	34 (57%)	46 (61%)	
Female	26 (43%)	29 (39%)	
**Race, n (%)**			χ^2^ = (6, *n =* 135) = 10.03, *p* = .12
American Indian or Alaskan Native	1 (1.7%)	1 (1.3%)	
Native Hawaiian or Pacific Islander	-	2 (2.7%)	
Asian	7 (11.7%)	4 (5.3%)	
Black or African American	4 (6.7%)	-	
More than one race	11 (18.3%)	12 (16%)	
Unknown or Declined to State	1 (1.7%)	4 (5.3%)	
White	36 (60%)	52 (69.3%)	
**Ethnicity, n (%)**			χ^2^ = (2, *n =* 135) = 0.37, *p* = .83
Hispanic or Latino	12 (20%)	12 (16%)	
Not Hispanic or Latino	45 (75%)	59 (79%)	
Unknown or Declined to State	3 (5%)	4 (5%)	
**Household income, n (%)**			χ^2^ = (5, *n =* 135) = 5.41, *p* = .37
< $25k	5 (8.3%)	1 (1.3%)	
$25k to $50k	2 (3.3%)	4 (5.3%)	
$51k to $80k	7 (11.7%)	10 (13.3%)	
$81k to $100k	9 (15%)	7 (9.3%)	
> $100k	30 (50%)	44 (59%)	
Unknown or Declined to State	7 (11.7%)	9 (12%)	

Parents of toddlers with lower likelihood (LL) of ASD (*n* = 88) also completed the M-CHAT-R. The LL sample was used as a comparison group to examine consistency in reporting of ASD symptoms across the M-CHAT-R and a developmental concerns interview. There were too few ASD diagnoses within the LL group (*n* = 3) to compare the M-CHAT-R’s screening properties (e.g., SE, SP, etc.) across the HL and LL groups. LL infants had typically developing older siblings confirmed by an intake interview and proband SCQ scores below the ASD cutoff. Exclusion criteria for both groups were birth before 32 weeks of gestation and a known genetic disorder in the proband. Parents provided informed consent and the study was approved by the university’s Institutional Review Board.

### Measures

Visits were conducted at a major medical center in the United States at 18, 24, and 36 months by examiners unaware of HL v. LL status, as well as results from previous visits.

*Modified-Checklist for Autism in Toddlers, Revised (M-CHAT-R;* Robins et al., 2014) is a parent-report checklist with 20 yes/no response items. A positive screen is defined as 3 or more failed items. The M-CHAT-R questionnaire was mailed to parents and completed prior to their in-person evaluation visits; for one participant, the M-CHAT-R was filled out at 24 months but the family did not attend the visit, resulting in missing diagnostic data for one child at 24 months. The follow-up interview was not conducted as part of this study.

*Parent Concerns Interview* (Ozonoff et al., 2009). At 18- and 24-months, parents were asked if they had concerns about their child’s behavior or development. Responses were classified by coders trained to 80% reliability into 1 of 10 categories of concern (none, social, language, repetitive behavior, motor, medical, temperament/behavior, general developmental, other, and unspecified ASD concerns). Primary variable was number of ASD-related concerns (a sum of social, language, repetitive behavior, and unspecified ASD concerns).

*Mullen Scales of Early Learning* (MSEL; Mullen, 1995) is a standardized developmental test for children birth to 68 months that measures motor, cognitive, and language skills. It has good internal, test-retest, and interrater reliability and convergent validity.

*Autism Diagnostic Observation Schedule-2 (ADOS-2;* Lord et al., 2012) is a semi-structured play-based interaction and observation that provides a comparison score (range 1–10) with a cutoff of 4 that distinguishes ASD from non-ASD cases (Gotham et al., 2009). The ADOS-2 was administered at 18, 24, and 36 months by examiners who had completed rigorous research training and achieved 80% or higher reliability with a trainer throughout the study.

*Diagnostic Classification.* After each visit (18, 24, and 36 months), a binary diagnostic classification (ASD and Non-ASD) was made. The ASD classification was defined as obtaining an ADOS-2 comparison score of 4 or higher and meeting *Diagnostic and Statistical Manual of Mental Disorders* 5th ed. (DSM-5; American Psychiatric Association, 2013) criteria for ASD, verified by a licensed clinician. All participants not meeting these criteria were classified as Non-ASD. This classification could change from visit to visit, as signs of ASD emerged over time and (rarely) early diagnoses were not confirmed at later ages. In the HL sample, *n* = 11 were classified as ASD at 18 months, *n* = 14 at 24 months, and *n* = 22 at 36 months.

*Statistical Analysis.* SE (detecting ASD when it is truly present) was calculated by dividing the number of True Positives (i.e., participants with an M-CHAT-R score of 3 or above who received an ASD diagnosis) by the total number of children with an ASD outcome (True Positives/(True Positives + False Negatives) x 100). SP (detecting non-ASD cases accurately) was calculated by dividing true negatives (i.e., participants with an M-CHAT-R score of 2 or below who did not receive an ASD diagnosis) by the total number of children with a Non-ASD outcome classification (True Negatives/(True Negatives + False Positives) x 100). PPV (proportion of positive screens diagnosed with ASD) was calculated by dividing True Positives by all screen positives (True Positives/(True Positives + False Positives) x 100) and NPV (proportion of negative screens with Non-ASD outcomes) was calculated by dividing True Negatives for ASD by all screen negatives (True Negatives/(True Negatives + False Negatives) x 100). Differences in psychometric values in the single versus repeat screening groups were tested using Fisher’s exact test of independence (McDonald, 2009). The AAP considers SE and SP above 70% to be acceptable for ASD-specific screening measures (Council on Children With Disabilities, Section on Developmental Behavioral Pediatrics, Bright Futures Steering Committee, 2006).

SE, SP, PPV, and NPV were calculated for two non-overlapping HL samples: a single screen group (*n* = 60, 18 months) and a repeat screen group (*n* = 75, 18 and 24 months). We examined the M-CHAT-R’s ability to concurrently distinguish ASD from Non-ASD at 18 and 24 months, as well as to predictively identify it at 36 months, comparing single versus repeat screenings.

To examine whether reporting of early symptoms differs across parents with and without familiarity with ASD, analyses were conducted using both HL and LL participants. Separate bivariate correlations were conducted to examine the concordance between parent-reported ASD concerns and M-CHAT-R scores at 18 and 24 months in the HL and LL groups. We used Fisher’s Z transformation to statistically compare the magnitude of the relationships within the two likelihood groups. Finally, a repeated measures analysis of variance (ANOVA) was performed to analyze the effect of age (18 and 24 months), likelihood group (HL vs. LL), and ASD-related parent concerns on total M-CHAT-R scores. Simple main effects and interactions were analyzed. All analyses were implemented in IBM SPSS Statistics Version 28.0 (*IBM SPSS Statistics for Windows*, 2021).

## Results

Preliminary analyses were conducted to examine whether there were any systematic differences between the HL participants who were screened only once and those screened twice. Demographic characteristics were similar across the single and repeat screened groups (Table 1), suggesting that the sample screened only at 18 months did not differ systematically from the sample screened twice.

Table 2 displays the psychometric properties of the M-CHAT-R at 18 and 24 months using concurrent diagnostic classifications. SE was higher at 18 than 24 months. SP and PPV were higher at 24 than 18 months, replicating previous research (Sturner, Howard, Bergmann, Stewart, et al., 2017). We then compared the M-CHAT-R’s ability to prospectively identify ASD at 36 months, comparing the single to the repeat screened group; see Table 3. SE and PPV were higher in the repeat screened group (SE: 89% v. 75% with single screening; PPV: 76% v. 50% with single screening), with only a small decrease in SP with re-screening (SP: 91% v. 95% with single screening), also replicating previous research.

**Table 2:** Psychometric properties of the M-CHAT-R at 18 and 24 months for concurrently identifying ASD and Non-ASD in the HL group (n = 135)

Age of M-CHAT-R Screening	Concurrent ASD outcome		Concurrent Non-ASD outcome		Sensitivity (95% CI)	Specificity (95% CI)	Positive Predictive Value (95% CI)	Negative Predictive Value (95% CI)
	**M-CHAT-R Score ≥ 3** **(True Positives)**	**M-CHAT-R Score ≤ 2** **(False Negatives)**	**M-CHAT-R Score ≥ 3** **(False Positives)**	**M-CHAT-R Score ≤ 2 (True Negatives)**				
**18-months** **(n = 135)**	10	1	13	111	91% (57–100%)	90% (82–94%)	43% (24–65%)	99% (94–100%)
**24-months** (n = 74)*	11	3	4	56	79% (49–94%)	93% (82–98%)	73% (45–91%)	95% (85–99%)

Note.*n = 75 participants had a 24-month M-CHAT-R screening but one missed their 24-month diagnostic evaluation, so their concurrent ASD outcome is missing

**Table 3:** Psychometric properties of the M-CHAT-R for single vs. repeat screenings for predictively distinguishing ASD from Non-ASD at 36 months for the HL group only (n = 135)

Age of M-CHAT-R Screening	ASD outcome at 36 months		Non-ASD outcome at 36 months		Sensitivity (95% CI)	Specificity (95% CI)	Positive Predictive Value (95% CI)	Negative Predictive Value (95% CI)
	**M-CHAT-R Score ≥ 3** **(True Positives)**	**M-CHAT-R Score ≤ 2** **(False Negatives)**	**M-CHAT-R Score ≥ 3** **(False Positives)**	**M-CHAT-R Score ≤ 2** **(True** **Negatives)**				
**Single Screening at 18-months** **(n = 60)**	3	1	3	53	75% (22–99%)	95% (84–99%)	50% (14–86%)	98% (89–100%)
**Repeat Screenings at 18-** ***and*** 24-months* **(n = 75)**	16	2	5	52	89% (64–98%)	91% (80–97%)	76% (52–91%)	96% (86–99%)

Note.*If ever screened positive

Finally, we examined the relationship between the total number of parent-reported ASD concerns and total M-CHAT-R scores at 18 and 24 months within each likelihood group (HL vs. LL). At both ages, the correlation coefficients for the HL group were higher than that for the LL group (18-months- HL: *r* = .45, *p* < .01; LL: *r* = .26, *p* < .05; 24-months- HL: *r* = .63, *p* < .01; LL: *r* = -.05 *p* > .05). The difference between the HL and LL correlation coefficients approached statistical significance (Fisher’s Z = 1.55; *p* = .06) at 18 months and was significantly different (Fisher’s Z = 3.75; *p* < .05) at 24 months.

**Fig. 1:**
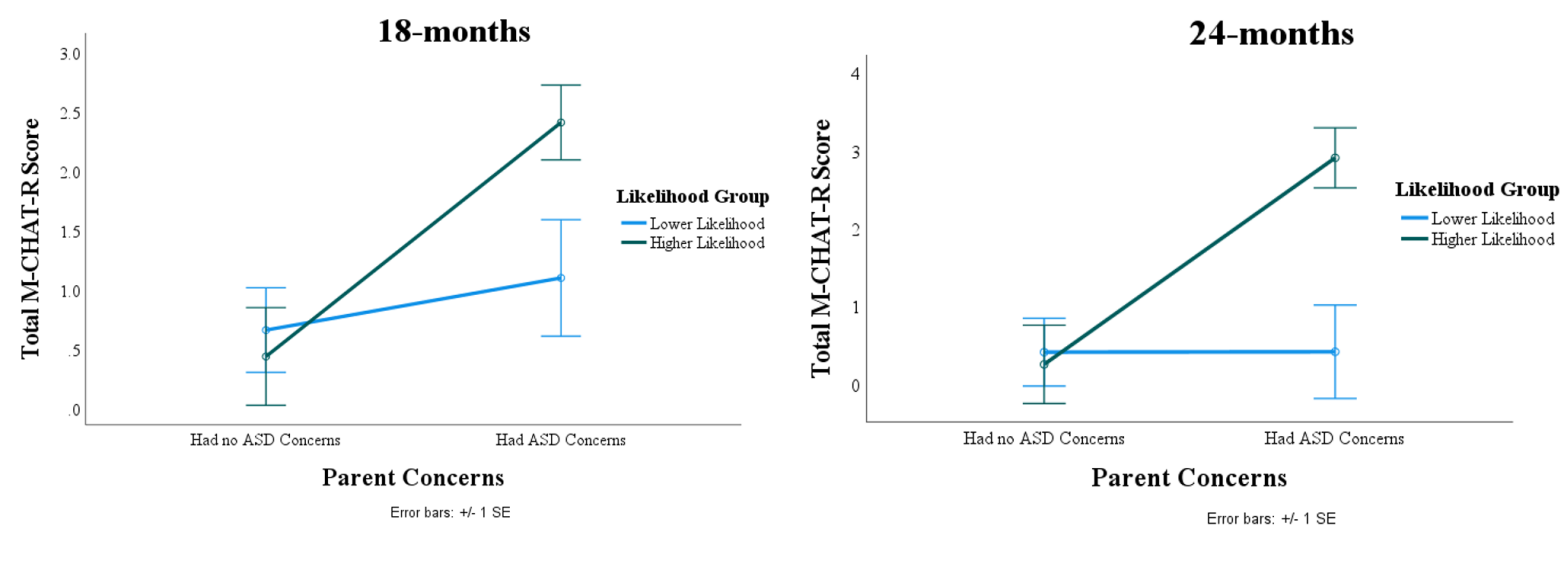
Total M-CHAT R scores plotted by likelihood group and the presence or absence of at least one ASD-related parent concern at 18 and 24 months

A repeated measures ANOVA was conducted to analyze the effects of age, likelihood group (HL vs. LL), and dichotomously-defined ASD-related parent concerns (if parents ever had ASD concerns vs. no ASD concerns) on total M-CHAT-R scores (see Table 4; Fig. 1). There was no statistically significant difference of age on total M-CHAT-R scores (*p* > .05). There were significant main effects on M-CHAT-R scores of both likelihood group (*p* < .05) and parent-reported ASD concerns (*p* < .01), and a significant interaction between likelihood group and ASD concerns (*F* (1, 124) = 6.63, *p* < .01, *p* < .01, ${\eta }_{p}^{2}=$0.05), indicating a small effect size (Cohen, 1988). Examination of simple effects demonstrated that the effect of ASD concerns depended on likelihood group. For the LL group, there was little difference in total M-CHAT-R scores between parents with and without ASD concerns. However, for the HL group, parents with ASD-related concerns had higher M-CHAT-R scores than those without ASD concerns (see Fig. 1).

**Table 4:** M-CHAT-R scores as a function of likelihood group, age, and parent concerns

*Descriptive Results*											
		**18-months Total M-CHAT-R**					**24-months Total M-CHAT-R**				
		*Mean*		*SD*			*Mean*			*SD*	
**Higher Likelihood Group**											
Parents had ASD concerns (n = 46)		2.41		3.25			2.91			4.23	
Parents had no ASD concerns (n = 27)		0.44		0.70			0.26			0.59	
**Lower Likelihood Group**											
Parents had ASD concerns (n = 19)		1.11		1.41			0.42			0.77	
Parents had no ASD concerns (n = 36)		0.67		1.10			0.42			0.81	
*Repeated Measures ANOVA Results*											
***Source of Variation***	***Sum of Squares***		***df***		***Mean Square***	***F***		***p-value***	***Partial Eta Squared (*** ${\varvec{\eta }}_{\varvec{p}}^{2})$		
Within Groups											
Age	1.38		1		1.38	0.71		0.40	0.01		
Age x Recruitment Group	5.60		1		5.60	2.89		0.09	0.02		
Age x Parent Concerns	0.23		1		0.23	0.12		0.73	0.00		
Age x Recruitment Group x Parent Concerns	4.50		1		4.50	2.32		0.13	0.02		
Error	240.22		124		1.94						
Between Groups											
Recruitment group	42.02		1		42.02	4.44		**0.04**	0.04		
Parent Concerns	92.17		1		92.17	9.74		**0.01**	0.07		
Recruitment Group x Parent Concerns	62.75		1		62.75	6.63		**0.01**	0.05		
Error	1173.61		124		9.47						

*Note.* η^2^ effect size benchmarks, η^2^ < 0.06 (small), η^2^ 0.06 to 0.13 (medium), and η^2^ ≥ 0.14 (large) (Cohen, 1988)

## Discussion

Many previously published studies of the M-CHAT-R did not have a comprehensive strategy for identifying missed cases among those who screened negative, following only those who screened positive and focusing on identification of false positives. This strategy, while more efficient and feasible for large-scale community screening, only permits calculation of PPV and not SE or SP (Levy et al., 2020; McPheeters et al., 2016; Sheldrick et al., 2023). The current study prospectively followed a large sample of HL toddlers, regardless of screening status, from initial screening at 18 months to final diagnostic classification at 36 months. This is one of only a few studies to follow both screen positives and all screen negatives to diagnostic outcome age, allowing more accurate estimation of all psychometric properties (SE, SP, PPV, NPV). We found high SE and SP (ranging from 75 to 95%) in this study that were in line with the pooled SE of 83% and SP of 94% reported in the meta-analysis of Wieckowski et al. (2023), which included studies with weaker methods for case confirmation among screen-negatives.

This is also one of only a handful of investigations (3 of 51 studies in a recent meta-analysis; Wieckowski et al., 2023) to compare the utility of single versus repeat screenings, finding a large increase in SE (89% v. 75%) with only a small decrease in SP (95% v. 91%) with repeated screening. This adds to the literature suggesting that repeated screenings, in line with AAP guidelines, may help reduce the age of ASD diagnosis (Guthrie et al., 2019; Hyman et al., 2020; Zwaigenbaum & Maguire, 2019). Repeat screening only modestly increased false positives, 60% of whom had other developmental concerns including high activity level and speech delays, and likely benefitted from additional evaluations. Non-autism developmental delays are common in siblings of children with ASD (Bradbury et al., 2020; Sacrey et al., 2015) and thus may have inflated the false positive rate in this study.

Finally, we explored differences in M-CHAT-R reporting across parents with more (HL group) versus less (LL group) knowledge of ASD, due to family history. We found higher concordance between the reporting of ASD concerns and M-CHAT-R screening in the HL group than the LL group, such that parents with an older child with ASD were more consistent in their reporting across the two measures. This suggests that M-CHAT-R scores may be more reflective of concerns in parents who have previous experience with ASD than in those with less familiarity with ASD. As was the case in previous HL samples (Bradbury et al., 2020; Weitlauf et al., 2015), the PPV estimates from the current HL sample (ranging from 43 to 76%) were much higher than the PPV of 14% reported in the M-CHAT-R validation sample (Robins et al., 2014). Collectively, these findings suggest that the M-CHAT-R may be particularly helpful for identifying possible ASD in toddlers with an older affected sibling.

There are several limitations to the current study. First, no M-CHAT-R follow-up interviews were conducted, which are known to reduce false positives and thereby improve PPV (Robins et al., 2014). Second, despite having a relatively large sample of HL toddlers, the overall sample size is much smaller than community screening studies. Finally, we were unable to directly compare psychometric properties of the M-CHAT-R in HL versus LL toddlers because too few participants in the LL group developed ASD.

The contributions of the present study include demonstration of the benefits of repeat screening and estimation of SE and SP in a large HL sample with diagnostic testing conducted regardless of screening status. This study design can identify cases missed by initial screening and thus provide more accurate calculations of SE and SP. Our results, using this comprehensive strategy to identify false negatives, are comparable to the pooled estimates of SE and SP for the M-CHAT-R recently reported in a meta-analysis (Wieckowski et al., 2023).

In conclusion, this data demonstrates the strong performance of the M-CHAT-R when completed by parents who already have a child with ASD, suggesting that providers should pay close attention to scores over the cutoff for toddlers in such families, making prompt referrals for diagnostic evaluation. Comprehensive developmental monitoring and repeated ASD screenings are recommended by the AAP for all children. While this study highlights their utility specifically for children at higher likelihood of ASD due to family history, regular and repeat screenings with the M-CHAT-R are also critical for children in the general population (Hyman et al., 2020), given the instrument’s ability to identify both autism and broader developmental concerns.

## Abbreviations

AAP: American Academy of Pediatrics
ADOS-2: Autism Diagnostic Observation Schedule-2
ASD: Autism spectrum disorder
CI: Confidence Interval
HL: Higher Likelihood
LL: Lower Likelihood
M-CHAT-R: Modified Checklist for Autism in Toddlers, Revised
MSEL: Mullen Scales of Early Learning
Non-ASD: Non-autism spectrum disorder outcome
NPV: Negative Predictive Value
PPV: Positive Predictive Value
SE: Sensitivity
SP: Specificity

## References

[CR1] American Psychiatric Association (2013). *Diagnostic and statistical manual of mental disorders (DSM-5®)*. 10.1590/s2317-17822013000200017 24413388

[CR2] Bradbury, K., Robins, D. L., Barton, M., Ibañez, L. V., Stone, W. L., Warren, Z. E., & Fein, D. (2020). Screening for autism spectrum disorder in high-risk younger siblings. *Journal of Developmental and Behavioral Pediatrics: JDBP*, *41*(8), 596. 32576788 10.1097/DBP.0000000000000827 PMC7572497

[CR3] Carbone, P. S., Campbell, K., Wilkes, J., Stoddard, G. J., Huynh, K., Young, P. C., & Gabrielsen, T. P. (2020). Primary care autism screening and later autism diagnosis. *Pediatrics*, *146*(2), e20192314. 10.1542/peds.2019-2314. 32632024 10.1542/peds.2019-2314 PMC7397730

[CR4] Cohen, J. (1988). Chapter 8. The analysis of variance and covariance. *Statistical power analysis for the behavioral Sciences; Routledge Academic* (pp. 273–406). New York, NY, USA.

[CR5] Council on Children With Disabilities, Section on Developmental Behavioral Pediatrics, Bright Futures Steering Committee, & Medical Home Initiatives for Children with Special Needs Project Advisory Committee. (2006). Identifying infants and young children with developmental disorders in the medical home: An algorithm for developmental surveillance and screening. *Pediatrics*, *118*(1), 405–420. 16818591 10.1542/peds.2006-1231

[CR6] Gotham, K., Pickles, A., & Lord, C. (2009). Standardizing ADOS scores for a measure of severity in autism spectrum disorders. *Journal of Autism and Developmental Disorders*, *39*(5), 693–705. 19082876 10.1007/s10803-008-0674-3 PMC2922918

[CR7] Guthrie, W., Swineford, L. B., Nottke, C., & Wetherby, A. M. (2013). Early diagnosis of autism spectrum disorder: Stability and change in clinical diagnosis and symptom presentation. *Journal of Child Psychology and Psychiatry*, *54*(5), 582–590. 23078094 10.1111/jcpp.12008 PMC3556369

[CR8] Guthrie, W., Wallis, K., Bennett, A., Brooks, E., Dudley, J., Gerdes, M., Pandey, J., Levy, S. E., Schultz, R. T., & Miller, J. S. (2019). Accuracy of Autism Screening in a large Pediatric Network. *Pediatrics*, *144*(4), e20183963. 10.1542/peds.2018-3963. 31562252 10.1542/peds.2018-3963

[CR9] Hyman, S. L., Levy, S. E., & Myers, S. M., Council on children with disabilities, section on developmental and behavioral pediatrics. (2020). Identification, evaluation, and management of children with Autism Spectrum Disorder. *Pediatrics*, *145*(1), e20193447. 10.1542/peds.2019-3447. 10.1542/peds.2019-3447 31843864

[CR10] *IBM SPSS Statistics for Windows* (28.0). (2021). IBM Corp.

[CR11] Levy, S. E., Wolfe, A., Coury, D., Duby, J., Farmer, J., Schor, E., Van Cleave, J., & Warren, Z. (2020). Screening tools for autism spectrum disorder in primary care: A systematic evidence review. *Pediatrics*, *145*(Supplement_1), S47–S59. 32238531 10.1542/peds.2019-1895H

[CR12] Lord, C., Rutter, M., DiLavore, P. C., Risi, S., Gotham, K., & Bishop, S. L. (2012). *Autism Diagnostic Observation schedule Manual* (2nd ed.). Western Psychological Services.

[CR13] Maenner, M. J., Warren, Z., Williams, A. R., Amoakohene, E., Bakian, A. V., Bilder, D. A., Durkin, M. S., Fitzgerald, R. T., Furnier, S. M., Hughes, M. M., Ladd-Acosta, C., McArthur, D., Pas, E. T., Salinas, A., Vehorn, A., Williams, S., Esler, A., Grzybowski, A., Hall-Lande, J., & Shaw, K. A. (2023). Prevalence and characteristics of autism spectrum disorder among children aged 8 years—Autism and Developmental Disabilities Monitoring Network, 11 sites, United States, 2020. *MMWR Surveillance Summaries*, *72*. 10.15585/mmwr.ss7202a1 PMC10042614 36952288

[CR14] McCarty, P., & Frye, R. E. (2020). Early detection and diagnosis of autism spectrum disorder: Why is it so difficult? *Seminars in Pediatric Neurology*, *35*, 100831. 32892958 10.1016/j.spen.2020.100831

[CR15] McDonald, J. H. (2009). Fisher’s exact test of independence. *Handbook of Biological Statistics*, *2*, 75–79.

[CR16] McPheeters, M. L., Weitlauf, A., Vehorn, A., Taylor, C., Sathe, N. A., Krishnaswami, S., Fonnesbeck, C., & Warren, Z. E. (2016). *Screening for autism spectrum disorder in young children: A systematic evidence review for the US Preventive Services Task Force*. 26985520

[CR17] Mullen, E. M. (1995). *Mullen Scales of early learning*. Circle Pines.

[CR18] Øien, R. A., Schjølberg, S., Volkmar, F. R., Shic, F., Cicchetti, D. V., Nordahl-Hansen, A., Stenberg, N., Hornig, M., Havdahl, A., Øyen, A. S., Ventola, P., Susser, E. S., Eisemann, M. R., & Chawarska, K. (2018). Clinical features of children with Autism who passed 18-Month Screening. *Pediatrics*, *141*(6), e20173596. 10.1542/peds.2017-3596. 29784756 10.1542/peds.2017-3596

[CR22] Ozonoff, S., Young, G. S., Steinfeld, M. B., Hill, M. M., Cook, I., Hutman, T., Macari, S., Rogers, S. J., & Sigman, M. (2009). How early do parent concerns predict later autism diagnosis? *Journal of Developmental and Behavioral Pediatrics: JDBP*, *30*(5), 367–375. 10.1097/dbp.0b013e3181ba0fcf. 19827218 10.1097/dbp.0b013e3181ba0fcf PMC2919345

[CR19] Ozonoff, S., Iosif, A. M., Baguio, F., Cook, I. C., Hill, M. M., Hutman, T., Rogers, S. J., Rozga, A., Sangha, S., Sigman, M., Steinfeld, M. B., & Young, G. S. (2010). A prospective study of the emergence of early behavioral signs of autism. *Journal of the American Academy of Child & Adolescent Psychiatry*, *49*(3), 256–266e2. 10.1016/j.jaac.2009.11.009. 20410715 PMC2923050

[CR21] Ozonoff, S., Young, G. S., Landa, R. J., Brian, J., Bryson, S., Charman, T., Chawarska, K., Macari, S. L., Messinger, D., & Stone, W. L. (2015). Diagnostic stability in young children at risk for autism spectrum disorder: A baby siblings research consortium study. *Journal of Child Psychology and Psychiatry*, *56*(9), 988–998. 25921776 10.1111/jcpp.12421 PMC4532646

[CR20] Ozonoff, S., Young, G. S., Brian, J., Charman, T., Shephard, E., Solish, A., & Zwaigenbaum, L. (2018). Diagnosis of Autism Spectrum Disorder after Age 5 in children evaluated longitudinally since infancy. *Journal of the American Academy of Child & Adolescent Psychiatry*. 10.1016/J.JAAC.2018.06.022. 10.1016/j.jaac.2018.06.022 PMC6235445 30392626

[CR23] Pierce, K., Gazestani, V. H., Bacon, E., Barnes, C. C., Cha, D., Nalabolu, S., Lopez, L., Moore, A., Pence-Stophaeros, S., & Courchesne, E. (2019). Evaluation of the diagnostic stability of the early autism spectrum disorder phenotype in the general population starting at 12 months. *JAMA Pediatrics*, *173*(6), 578–587. 31034004 10.1001/jamapediatrics.2019.0624 PMC6547081

[CR25] Robins, D. L., Fein, D., Barton, M. L., & Green, J. A. (2001). The modified checklist for Autism in Toddlers: An initial study investigating the early detection of autism and pervasive developmental disorders. *Journal of Autism & Developmental Disorders*, *31*(2), 131. 11450812 10.1023/a:1010738829569

[CR24] Robins, D. L., Casagrande, K., Barton, M., Chen, C. M. A., Dumont-Mathieu, T., & Fein, D. (2014). Validation of the modified checklist for autism in toddlers, revised with follow-up (M-CHAT-R/F). *Pediatrics*, *133*(1), 37–45. 24366990 10.1542/peds.2013-1813 PMC3876182

[CR26] Rutter, M., Bailey, A., & Lord, C. (2003). *The social communication questionnaire: Manual*. Western Psychological Services.

[CR27] Sacrey, L. A. R., Zwaigenbaum, L., Bryson, S., Brian, J., Smith, I. M., Roberts, W., Szatmari, P., Roncadin, C., Garon, N., Novak, C., Vaillancourt, T., McCormick, T., MacKinnon, B., Jilderda, S., & Armstrong, V. (2015). Can parents’ concerns predict Autism Spectrum Disorder? A prospective study of high-risk siblings from 6 to 36 months of age. *Journal of the American Academy of Child & Adolescent Psychiatry*, *54*(6), 470–478. 10.1016/j.jaac.2015.03.014. 26004662 10.1016/j.jaac.2015.03.014

[CR28] Sheldrick, R. C., Hooker, J. L., Carter, A. S., Feinberg, E., Croen, L. A., Kuhn, J., Slate, E., & Wetherby, A. M. (2023). The influence of loss to follow-up in autism screening research: Taking stock and moving forward. *Journal of Child Psychology and Psychiatry*, online ahead of print, https://10.1111/jcpp.13867. 10.1111/jcpp.13867 PMC10801774 37469104

[CR30] Sturner, R., Howard, B., Bergmann, P., Stewart, L., & Afarian, T. E. (2017). Comparison of autism screening in younger and older toddlers. *Journal of Autism and Developmental Disorders*, *47*(10), 3180–3188. 28733850 10.1007/s10803-017-3230-1 PMC5711534

[CR29] Sturner, R., Howard, B., Bergmann, P., Morrel, T., Landa, R., Walton, K., & Marks, D. (2017a). Accurate autism screening at the 18-month well-child visit requires different strategies than at 24 months. *Journal of Autism and Developmental Disorders*, *47*(10), 3296–3310. 10.1007/s10803-017-3231-0. 28762159 10.1007/s10803-017-3231-0 PMC5711554

[CR31] Toh, T. H., Tan, V. W. Y., Lau, P. S. T., & Kiyu, A. (2018). Accuracy of modified Checklist for Autism in Toddlers (M-CHAT) in detecting autism and other developmental disorders in community clinics. *Journal of Autism and Developmental Disorders*, *48*(1), 28–35. 10.1007/s10803-017-3287-x. 28866856 10.1007/s10803-017-3287-x

[CR32] Weitlauf, A. S., Vehorn, A. C., Stone, W. L., Fein, D., & Warren, Z. E. (2015). Using the M-CHAT-R/F to identify developmental concerns in a high-risk 18-month-old sibling sample. *Journal of Developmental and Behavioral Pediatrics: JDBP*, *36*(7), 497. 26171765 10.1097/DBP.0000000000000194 PMC4564349

[CR33] Wieckowski, A. T., Williams, L. N., Rando, J., Lyall, K., & Robins, D. L. (2023). Sensitivity and specificity of the modified checklist for Autism in Toddlers (original and revised): A systematic review and Meta-analysis. *JAMA Pediatrics*. 10.1001/jamapediatrics.2022.5975 PMC9941975 36804771

[CR34] Yuen, T., Carter, M. T., Szatmari, P., & Ungar, W. J. (2018). Cost-effectiveness of universal or high-risk screening compared to surveillance monitoring in autism spectrum disorder. *Journal of Autism and Developmental Disorders*, *48*(9), 2968–2979. 29644584 10.1007/s10803-018-3571-4

[CR36] Zwaigenbaum, L., & Maguire, J. (2019). Autism screening: Where do we go from here? *Pediatrics*, *144*(4). 10.1542/peds.2019-0925 31562253

[CR35] Zwaigenbaum, L., Bryson, S., & Garon, N. (2013). Early identification of autism spectrum disorders. *Behavioural Brain Research*, *251*, 133–146. 10.1016/j.bbr.2013.04.004. 23588272 10.1016/j.bbr.2013.04.004

